# Cascading impacts of the Maunder Minimum on rainfall and society in the Joseon dynasty

**DOI:** 10.1093/nsr/nwaf283

**Published:** 2025-07-12

**Authors:** Yuqi Wang, Yong Wei, Feng Shi, Xichen Li, Zhonghua Yao, Limei Yan, Yaochen Yue, Shiling Yang, Wei Lin, Yongxin Pan, Zhengtang Guo

**Affiliations:** Key Laboratory of Planetary Science and Frontier Technology, Institute of Geology and Geophysics, Chinese Academy of Sciences, Beijing 100029, China; Key Laboratory of Planetary Science and Frontier Technology, Institute of Geology and Geophysics, Chinese Academy of Sciences, Beijing 100029, China; College of Earth and Planetary Sciences, University of Chinese Academy of Sciences, Beijing 100049, China; State Key Laboratory of Lithospheric and Environmental Coevolution, Institute of Geology and Geophysics, Chinese Academy of Sciences, Beijing 100029, China; International Center for Climate and Environment Sciences, Institute of Atmospheric Physics, Chinese Academy of Sciences, Beijing 100029, China; Institute of Oceanography, Peking University, Beijing 100871, China; Department of Earth Sciences, The University of Hong Kong, Hong Kong 999077, China; Key Laboratory of Planetary Science and Frontier Technology, Institute of Geology and Geophysics, Chinese Academy of Sciences, Beijing 100029, China; College of Earth and Planetary Sciences, University of Chinese Academy of Sciences, Beijing 100049, China; Key Laboratory of Planetary Science and Frontier Technology, Institute of Geology and Geophysics, Chinese Academy of Sciences, Beijing 100029, China; College of Earth and Planetary Sciences, University of Chinese Academy of Sciences, Beijing 100049, China; College of Earth and Planetary Sciences, University of Chinese Academy of Sciences, Beijing 100049, China; State Key Laboratory of Lithospheric and Environmental Coevolution, Institute of Geology and Geophysics, Chinese Academy of Sciences, Beijing 100029, China; Key Laboratory of Planetary Science and Frontier Technology, Institute of Geology and Geophysics, Chinese Academy of Sciences, Beijing 100029, China; College of Earth and Planetary Sciences, University of Chinese Academy of Sciences, Beijing 100049, China; Key Laboratory of Planetary Science and Frontier Technology, Institute of Geology and Geophysics, Chinese Academy of Sciences, Beijing 100029, China; College of Earth and Planetary Sciences, University of Chinese Academy of Sciences, Beijing 100049, China; College of Earth and Planetary Sciences, University of Chinese Academy of Sciences, Beijing 100049, China; State Key Laboratory of Lithospheric and Environmental Coevolution, Institute of Geology and Geophysics, Chinese Academy of Sciences, Beijing 100029, China

**Keywords:** solar activity, Maunder Minimum, Joseon dynasty, rainfall reconstruction, extreme climate

## Abstract

Human civilization's evolution is shaped by climate change, with solar energy input into the Earth's system as the primary external driver. This influence should be more pronounced during agricultural stages and periods of extreme solar activity. The late Joseon Dynasty of Korea serves as an ideal civilization sample of political continuity and stability, maintaining a 285-year-long meteorological diary and rainfall records with a temporal resolution of up to 2 hours, perfectly encompassing the Maunder Minimum (MM). Here we quantitatively reconstruct the rainfall patterns, revealing a rare, nearly century-long drought around the MM, accompanied by decadal climate fluctuations correlated to the sunspot cycle. Quantitative socio-environmental analyses further indicate that the convergence of cold, arid conditions and heightened climate instability ultimately precipitated cascading ecological and societal crises during the late Joseon Dynasty. Our findings offer new perspectives for understanding and addressing the impacts of future periods of extreme solar activity on modern civilization.

## Teaser

A century-long drought during the Maunder Minimum, coupled with heightened climate instability linked to solar cycles, triggered cascading ecological and societal crises in Korea's late Joseon Dynasty.

## INTRODUCTION

Understanding the elusive relation between solar activity, climate change, and human civilization has long been a shared challenge for both natural and social sciences [1]. Solar activity modulates Earth's climate across diverse temporal and spatial scales through long-term variations in solar irradiance and short-term eruptive events [2,3]. These climatic shifts, in turn, affect agricultural productivity and population dynamics, profoundly shaping the trajectory of human societal development [4,5]. Because disentangling climate-induced societal crises from those driven by human activities remains challenging, the better choice is to study historical periods with less human interference before the industrial era, especially the periods of extreme solar activity.

The Maunder Minimum (MM, 1645–1715 CE) [6], the most pronounced grand minimum of solar activity since the advent of sunspot observations, offers a unique opportunity to examine the solar–climate–civilization relationship. Such extreme conditions could maximize the amplification of effective solar signals, thereby enhancing the sensitivity of both climate and societal responses [7–9]. The MM was characterized by pronounced cooling in the Northern Hemisphere [9] and widespread societal crises, including famines, epidemics, and social unrest in Europe and East Asia [10,11]. These findings exhibit significant nonlinearity and regional variability, highlighting the complex dynamics at play. Such complexities underscore the necessity of carefully contextualizing the characteristics of the selected civilizations and emphasizing the spatiotemporal heterogeneity of solar forcing when exploring the solar–climate–civilization relationship.

The Joseon Dynasty of Korea (1392–1910 CE) represented the final feudal dynasty on the Korean Peninsula and constituted a critical transitional period for East Asian civilization from traditional to modern society. This dynastic epoch completely spanned the MM. Yet the specific climatic impacts of this period of severely diminished solar activity on the Joseon Dynasty, and the role these climate-driven changes may have played in the dynasty's ultimate decline, remain poorly understood and have not been systematically examined. Therefore, we examine the late Joseon Dynasty as an ideal case study to investigate the impacts of extreme solar activity on climate change and subsequent societal responses. This ideality arises from the following factors: (i) a stable socio-political structure: the late Joseon period (1639–1910 CE) was a post-war recovery phase following foreign invasions. During this time, political transitions adhered to peaceful factional power shifts, with no major wars. This rare example of a self-contained, stable civilization allows the societal impacts of climate change to be observed with minimal interference from external factors such as dynastic transitions, invasions, or conflicts. (ii) High sensitivity of societal development to climate change: as a typical agrarian society, the Joseon economy after the 17th century was heavily dependent on rice cultivation. Rice, as a warm-season crop with a growing period extending from spring through autumn, exhibits high vulnerability to extreme climatic events. Rice yields are highly sensitive to both rainfall deficits and surpluses once seasonal totals depart from the local optimum [12]. Such sensitivity was markedly amplified in pre-industrial contexts where sophisticated irrigation infrastructure remained largely undeveloped. However, the scarcity of quantitative rainfall records has constrained the accuracy of existing reconstructions in capturing the region's rainfall during the MM, making this a key focus of our study. (iii) Comprehensive historical records: the Joseon Dynasty's official annals (*Annals of the Joseon Dynasty*) and its foundational materials (*Seungjeongweon Ilgi*) provide detailed and continuous documentation of weather patterns, agricultural conditions, and societal events with annual resolution from the 17th to 19th centuries [13–15]. These records offer invaluable data for analyzing the climatic and societal impacts of the MM.

## RESULTS

### Prolonged drought during the Maunder Minimum

**Figure 1.**
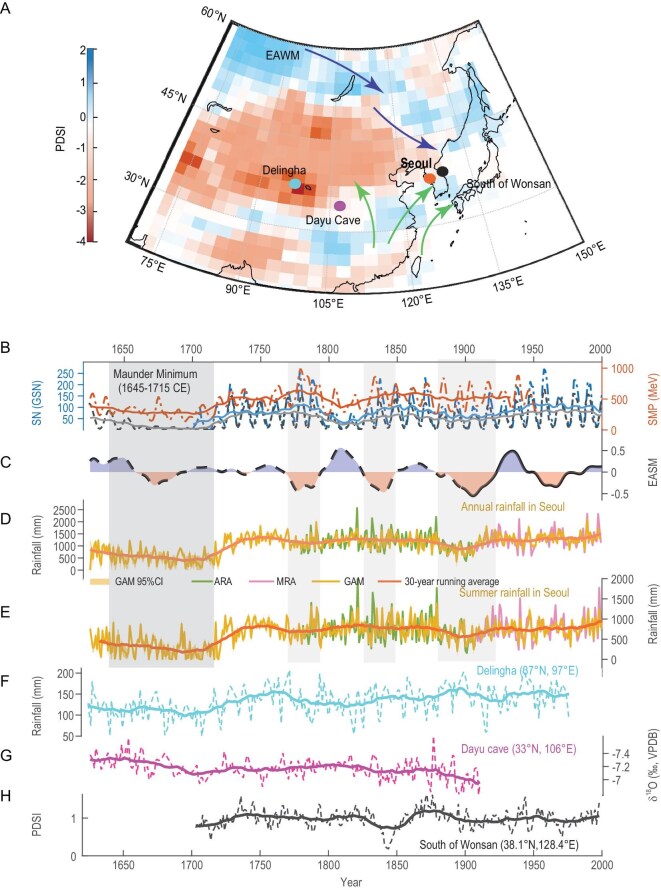
Reconstructed rainfall for Seoul (this study) and East Asia. (A) Study region and distribution of rainfall data points. Background indicates the average Palmer Drought Severity Index (PDSI) of East Asian summer (June–August) during 1645–1715 CE, as obtained from Paleo Hydrodynamics Data Assimilation products (PHYDA). (B) Annual variations in solar activity and their 30-year moving averages. The red curve represents the solar modulation potential (SMP) reconstructed from tree-ring ^14^C records [20]. The grey curve shows the reconstructed group sunspot number (GSN) [21]. The blue curve indicates the yearly mean total sunspot number (SN) from 1700 to the present (Source: WDC-SILSO, Royal Observatory of Belgium, Brussels). (C) Decadal variations in the EASM index [17]. The dashed line represents EASM reconstructions based on climate proxy data, while the solid line shows reconstructions using modern instrumental measurements. Blue shading highlights positive EASM values, while pink shading indicates negative values. (D) GAM-based reconstruction of annual rainfall amounts for Seoul (yellow curve) and its 30-year moving average (orange bold curve). The yellow shading indicates the 95% confidence interval (95% CI) of the reconstruction. The green curve represents observed ancient rainfall amounts (ARA) [15], and the pink curve shows observed modern rainfall amounts (MRA). (E) Similar to panel D, but showing reconstructed summer rainfall amounts (June–August). (F) Annual rainfall reconstructed from tree-ring series in the Delingha. (G) Speleothem δ^18^O variations from Dayu Cave, where lower values indicate higher rainfall. (H) The annual PDSI index reconstructed from tree-ring data in South Wonsan (38.1°N, 128.4°E) in northeastern Korea [18].

Seoul (37°33′N, 126°58′E), located in the midwestern region of the Korean Peninsula (Fig. 1A), experiences a temperate monsoon climate characterized by pronounced seasonal variability [12]. The majority of annual precipitation occurs during summer, driven by the East Asian Summer Monsoon (EASM), which transports warm, moist air from the ocean to the Korean Peninsula due to differential heating between land and ocean. In winter, a strong high-pressure system over the Siberian continent generates clockwise circulation, intensifying the East Asian winter monsoon (EAWM), which results in the advection of cold, dry air southeastward across the peninsula.

Ancient Korea was among the earliest regions in the world to conduct quantitative rainfall observations. The rainy-day records spanning 1625–1910 CE, extracted from daily weather documentation in the *Seungjeongweon Ilgi* (*Diary of the Royal Secretariat*), provide the only continuous rainfall dataset in the world that extends back to the MM (1645–1715 CE). The rainy-day records are formatted in the Gregorian calendar year-month-day format [13,14], while the quantitative rainfall records include the Gregorian calendar year-month-day along with detailed information on rainfall, including the timing and amount of rain [13,15]. We have assessed the scientific quality of these data to ensure their authenticity and coherence (see Methods). Based on the two datasets [14,15], we employed a generalized additive model (GAM) to reconstruct quantitative annual rainfall and summer rainfall (June–August) since 1625 CE, while quantifying the contributions of various modes of internal climate variability to the reconstruction. The model passed both the residual test and the statistical fidelity test, proving the reliability of rainfall reconstruction (see Methods). To evaluate the accuracy of the reconstruction (Fig. 1D and E), we compared the variance ratio and correlation between the reconstructed and observed rainfall datasets from 1780 CE to the present (Table S1). The annual correlation coefficients between measured and reconstructed rainfall amounts exceed 0.7 at the 99.9% confidence level, indicating that the reconstruction reliably captures interannual variations in both annual and summer rainfall. The variance ratio between measured and reconstructed rainfall amounts ranges from 1.33 to 1.36, suggesting that the reconstructed data effectively reflect the overall rainfall variability, though some extreme high and low rainfall events might be underestimated.

The most prominent decadal feature of Seoul's rainfall variability over the past 400 years is the prolonged drought during the MM. Figure 1B illustrates reconstructions of solar activity based on three different proxies. During the MM, average summer rainfall in Seoul dropped to 266 mm, accounting for just 31% of the mean summer rainfall over the past 70 years. Notably, the weather record was continuous between 1706 and 1707 CE, but no rainfall was documented in Seoul, thus effectively reducing annual rainfall to zero at the peak of this prolonged drought. The widespread 1707 drought was also recorded by speleothem isotopes and ancient stele inscriptions in Dayu Cave located on the southern slope of the Qinling Mountains in central China (33°N, 106°E) [16]. A Pettitt test identified a significant shift in both annual and summer rainfall in 1723 CE (Fig. S1), marking the abrupt end of the century-long drought and the onset of a rapid rainfall increase. Due to the continuous original rainfall records and the consistent reconstruction methods used, the significant changes in rainfall during and after the MM are likely not due to data merging or methodological shifts. Instead, they most likely reflect variations in regional circulation or changes in the intensity and path of EASM. Summer rainfall in East Asia is primarily influenced by the EASM. Strong EASM phases are typically associated with increased rainfall in northern China and the northern Korean Peninsula, whereas weak EASM phases result in enhanced rainfall near the Yangtze River basin. Long-term variations in the EASM index (Fig. 1C), reconstructed from tree rings, speleothems, and historical documents [17], indicate a general weakening of the EASM during the MM, accompanied by low variability. This subdued monsoon state is closely linked to reduced summer rainfall in Seoul.

To provide a more comprehensive understanding of regional precipitation characteristics, we introduce the Paleo Hydrodynamics Data Assimilation (PHYDA), which offers reconstructions of global hydroclimate over the past two millennia by integrating paleoclimate proxy time series data with the physical constraints of an atmosphere-ocean climate model [18]. The PHYDA hydrological reconstructions indicate that East Asian summer averaged rainfall during the MM exhibited tripolar rainfall heterogeneous patterns, characterized by increased rainfall in the middle and lower reaches of the Yangtze River, extending into southern Korea and southern Japan. In contrast, regions such as southern and southwestern China, northern China, and central East Asia experienced intensified drought conditions (Fig. 1A). Summer rainfall across the Korean Peninsula, particularly in the northern region, significantly decreased during the MM (Fig. S2). Although there are no quantitative rainfall proxy references for the Korean Peninsula before the 18th century, tree-ring rainfall reconstructions from South Wonsan (38.1°N, 128.4°E) clearly show a transition from drought to wet conditions in northeastern Korea during the early 18th century (Fig. 1H). This reconstruction show a strong decadal correlation coefficient of 0.8 (*p* <0.001) with rainfall records from Seoul (Fig. S3). The Delingha region in northwestern China (37°N, 97°E), located in the core area of this drought, also shows annual rainfall reconstructions from multiple tree-ring series [19], indicating drought and recovery phases similar to those observed in Seoul around the MM (Fig. 1F). Additionally, the speleothem δ^18^O record from Dayu Cave (33°N, 106°E) reveals a rapid reduction in rainfall during the MM (Fig. 1G) [16].

We further performed empirical mode decomposition (EMD) on the rainfall sequences at each location (Fig. S3). The results show that the IMF5 component, which accounts for ∼7%–10% of the explained variance in the original signal, indicates a significant reduction in rainfall starting in the mid-17th century, followed by a recovery in the mid-18th century. The IMF4 component, which explains about 6%–16% of the variance in the original signal, reveals that the rainfall signals in northern Korea and the inland areas of northern China exhibit consistent decadal oscillations with periods ranging from 20 to 60 years prior to the 19th century. This cross-regional rainfall correlation is likely the result of multiple large-scale circulation systems influenced by ocean-atmosphere interactions and external forcing [22].

### Enhanced solar cycle signal in rainfall variability

The EASM, which primarily controls rainfall in the East Asian region, exhibits significant variability on multidecadal timescales. Before the Industrial Revolution, multidecadal regional rainfall variability in East Asia was driven by a complex interplay of external forcings and internal climate variability. Internal climate variability, including the Pacific Decadal Oscillation (PDO) and the Atlantic Multidecadal Oscillation (AMO), can alter rainfall patterns in East Asia through ocean-atmosphere interactions [23,24]. External climate forcings are mainly associated with changes in solar activity and volcanic eruptions. Changes in solar irradiance altered land-sea temperature contrasts, driving changes in regional atmospheric circulation and consequently reshaping rainfall patterns [25]. Historical evidence from major eruptions, such as the Changbaishan volcano at the China–North Korea border, demonstrates that volcanic activity can significantly cool mid-to-high latitude regions for several years, with specific impacts on East Asian precipitation including increased rainfall in China's Meiyu belt while reducing precipitation over India and the South China Sea region [26].

Over the past 400 years, seven major global volcanic eruptions have been recorded, including six strong tropical eruptions and one in the Northern Hemisphere [27]. Two of these eruptions occurred near the MM (1641 and 1695 CE, both tropical), while the remaining five took place from the late 18th century to the mid-19th century (Fig. S4). We used the Superposed Epoch Analysis to evaluate the impact of seven major volcanic eruptions on annual rainfall in Seoul. The results show that strong global volcanic eruptions did not have a significant effect on rainfall in Seoul (Fig. S5). Additionally, we examined the eruption of Mount Changbai (41°N, 128°E) in June 1668, located near the China–Korea border and one of the closest volcanoes to our study site in Seoul. In the entire summer following this eruption, Seoul recorded only one rainfall event (July 18), marking the lowest summer rainfall total in a decade. The volcanic impact gradually diminished over the subsequent 2–3 years (Fig. S5). Notably, the MM did not coincide with a clustering of major volcanic eruptions, and volcanic forcing during this period primarily manifested as short-term intermittent impacts. Thus, no compelling evidence supports the attribution of the prolonged drought in 17th-century Seoul to volcanic activity.

**Figure 2.**
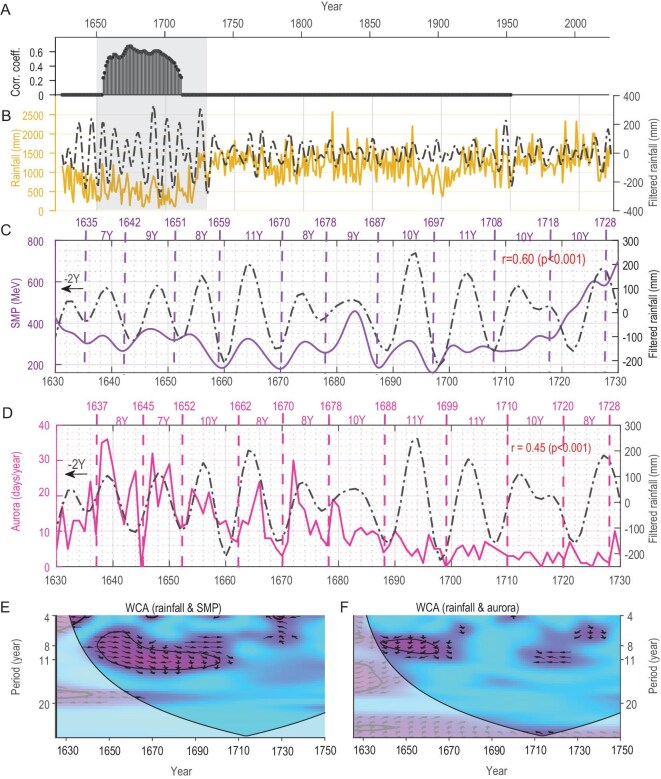
Correlation analysis between solar activity cycles and Seoul rainfall cycles during the Maunder Minimum. (A) Seventy-year moving correlation coefficients (*p* < 0.05) between solar modulation potential (SMP) and annual rainfall in Seoul, calculated with a 70-year sliding window and a 1-year step. (B) Annual rainfall in Seoul (yellow curve) and its 6–15-year bandpass-filtered variation (black dashed line). (C) SMP [20] with solar cycles defined by its minima (purple solid and dashed lines). The black dashed line represents Seoul's annual rainfall shifted forward by two years after applying a 7–15-year bandpass filter. (D) Historical aurora observations in Seoul [28] with solar cycles defined by the minima of annual auroral frequencies (pink solid and dashed lines). (E) Wavelet coherence analysis (WCA) between SMP and bandpass-filtered rainfall variations. Arrow length indicates the coherence strength between the two variables, while arrow direction represents their phase relationship: rightward arrows indicate complete in-phase coherence, and leftward arrows indicate complete anti-phase coherence. Bold black contours denote the 95% significance level. (F) Similar to panel E, but showing the WCA between annual auroral frequencies in Seoul and bandpass-filtered rainfall variations.

Solar forcing exhibits distinct characteristics compared to volcanic forcing, offering independent and robust constraints for identifying climate variability across different timescales. The most prominent quasi-periodic solar variation is the Schwabe cycle (sunspot cycle), which spans 8–14 years with an average period of ∼11 years and reflects the periodicity of the Sun's internal magnetic field. By applying a 7–15-year bandpass filter to the annual rainfall series in Seoul, we extracted rainfall signals corresponding to the Schwabe cycle. The results reveal a significant amplification of decadal rainfall variability during the MM (Fig. 2B). The average annual rainfall in Seoul during the MM was 502 mm, with decadal-scale rainfall variability averaging ∼120 mm. Consequently, decadal rainfall changes accounted for >20% of total rainfall variability during this period.

We further examined the relationship between rainfall variability and solar activity during the MM. Two independent annual-resolution datasets were used to represent solar activity: the solar modulation potential reconstructed from tree-ring ^14^C records [20] and the frequency of auroral sightings in Seoul [28]. Both datasets show that the periods of lowest solar activity, as defined by minima in solar modulation potential and auroral frequency, were closely aligned with the phases of decadal rainfall variability (Fig. 2C and D). This suggests that decadal rainfall fluctuations in Seoul were likely dominated by solar cycles during this period. Wavelet coherence analysis (WCA) further demonstrates a significant periodic correlation between solar modulation potential and decadal rainfall during the MM (Fig. 2E). This coherence exhibited finer structures, with shorter periods (∼8 years) during the early MM (1645–1670 CE) and longer periods (∼11 years) during the late MM (1670–1710 CE). Notably, this periodic correlation between solar activity and rainfall disappeared after the MM. Auroral frequency in Seoul also displayed significant coherence with decadal rainfall during the early MM, though this relationship weakened in the late MM due to sparse auroral data (Fig. 2F). Additionally, a 50-year sliding window correlation analysis (1-year step) between annual rainfall and solar modulation potential indicates that significant positive correlations were confined to the MM, while no significant correlation was observed outside periods of extreme solar minima (Fig. 2A, Fig. S6). These findings suggest that the influence of solar forcing on rainfall variability is temporally heterogeneous. During extreme periods of solar activity, such as the MM, the regulatory influence of solar activity on climate may become enhanced, resulting in unusual and pronounced fluctuations in climate records that exhibit a linear correlation with the solar cycle.

**Figure 3.**
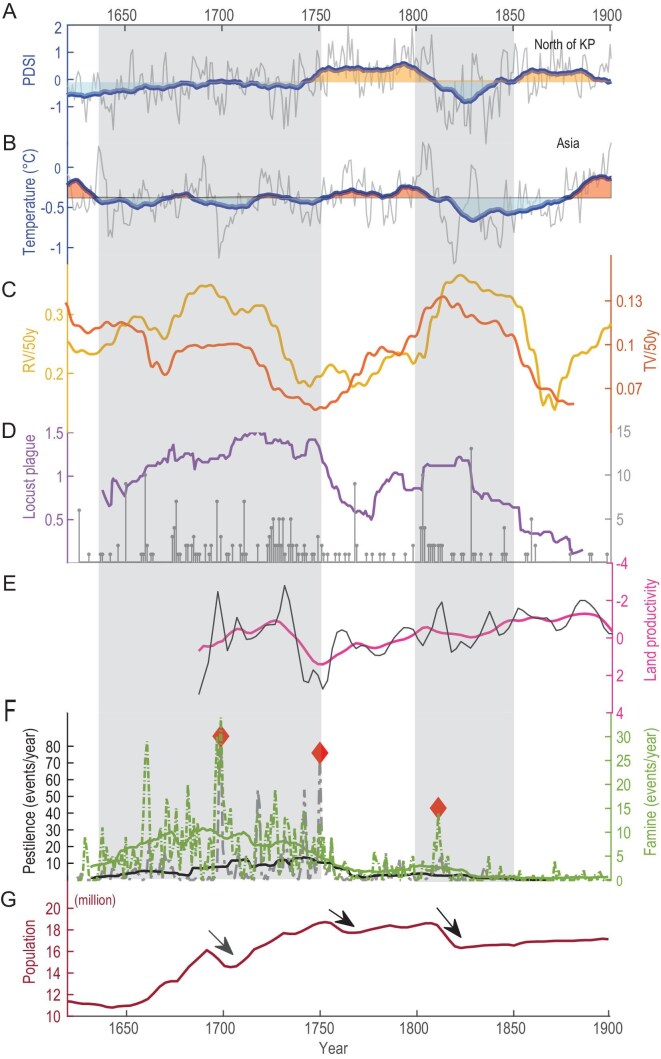
Climate variability and associated societal responses during the late Joseon dynasty. (A) Summer (June–August) Palmer Drought Severity Index (PDSI) reconstructed using Paleo Hydrodynamics Data Assimilation products (PHYDA) for the northern Korean Peninsula (KP, 38°N–43°N, 124°E–131°E) [18]. (B) Reconstructed surface temperature for Asia [29]. (C) Rainfall and temperature variability (RV/TV) for the northern Korean Peninsula and Asia, represented by the yellow and orange curves, respectively. (D) Annual frequency of locust outbreaks (gray matchstick plot) and their 30-year moving average (purple curve). (E) Land productivity index estimated from changes in land rental prices [36]. (F) Annual frequency of famine and pestilence events, along with their 30-year moving averages (green and black curves, respectively). (G) Population index during the late Joseon dynasty based on the dynastic Household Survey [33]. Solid arrows indicate the periods of rapid population decline.

We expanded the analysis to the northern Korean Peninsula, where regional climate reconstructions [18,29] reveal two distinct periods of concurrent drought and cold over the past 400 years (Fig. 3A and B): one from the mid-17th century to around 1750 CE (around the MM), and another from ∼1800 to 1850 CE (around the Dalton Minimum, during which climate changes are thought to have been primarily driven by volcanic forcing [30]). In the mid-to-late 17th century, summer temperatures across the Korean Peninsula declined significantly, as evidenced by an increased frequency of frost events and delayed last frost dates. Notably, four late frost events occurred during the summer months of June and July (Fig. S7) [31]. To assess climate variability across different timescales, we quantified rainfall and temperature variability (RV and TV) as the standard deviation of detrended rainfall and temperature over a 50-year moving window with a 1-year step (Fig. S8). The results indicate that periods of drought and cold coincided with significantly increased decadal climate variability (Fig. 3C). Recent studies on global population dynamics suggest that while societal adaptation to climate change weakens the correlation between population trends and mean climate states, population fluctuations remain strongly influenced by climate stability [1]. We will next explore the challenges faced by Joseon Dynasty society under such a climate condition characterized by drought, cold, and heightened instability.

### Cascading impacts from solar forcing to societal challenges

We reconstructed the frequency of locust outbreaks, famines, and pestilence from 1625 to 1910 CE using records of major livelihood events reported by local officials to the king during the Joseon Dynasty (see Methods). The results reveal two distinct peaks in locust activity from a long-term perspective: the mid-17th century to 1750 CE and 1800–1850 CE, both corresponding to the periods of cold and arid climatic conditions (Fig. 3D). While the overall frequency shows broad patterns, the most severe locust events provide clearer evidence of climate-society linkages. Extreme locust disaster events (defined as years with >5 locust outbreak records) occurred eight times: in 1650, 1661, 1676, 1697, 1711, 1768, 1803, and 1828 CE. Notably, five of these extreme events (62.5%) occurred during the MM, while two occurred during 1800–1850 CE. This pattern suggests that while moderate locust activity may not consistently align with drought conditions, the most severe outbreaks show a stronger correspondence with periods of climatic stress during solar minima.

Adverse climatic conditions and pest infestations reduced agricultural productivity, ultimately triggering widespread famine and epidemics. Rental prices for paddy fields during the late Joseon period (Fig. 3E) exhibited a sharp decline and increased volatility between 1690 and 1740 CE, reflecting reduced agricultural productivity rather than structural changes in tenancy systems [32]. Historical records document clustered outbreaks of famine and disease from the mid-17th century to 1750 CE, with the Great Famine of 1695–1699 CE representing the most severe crisis (Fig. 3F). Prolonged famine weakened immune defenses, facilitating the rapid spread of epidemics and resulting in over one million deaths nationwide. The *Annals of the Joseon Dynasty* recorded in October 1697: ‘This year, famine struck the entire country, with Gyeonggi (the region encompassing Seoul) and Chungcheong Province (southwestern Korea) being particularly severe. Corpses piled up in the capital’ (original record available at: https://sillok.history.go.kr/id/ksa_12310023_004).

Based on records from the dynastic Household Survey [33], the population of late Joseon Korea, despite an overall growth trend, experienced three distinct periods of rapid decline: 1695–1700 CE, 1750–1760 CE, and 1810–1816 CE. These population declines coincided with major outbreaks of famine and epidemics (Fig. 3F, 3G). Notably, our analysis reveals a significant inverse correlation between famine and epidemic indices and the 11- and 22-year solar cycles, suggesting that solar variability may have exerted a measurable influence on human societies (Fig. S9).

It is important to note that societal responses to long-term climate change are not instantaneous, but rather represent a prolonged process of dynamic adjustment. Ecological and agricultural systems require time to respond to cumulative climatic stress, while human activities such as deforestation and agricultural intensification, as well as political and economic conditions including ecological governance and food storage systems, can either amplify or buffer climate impacts.

We tend to believe that the current rainfall assimilated/reconstructed datasets [18] may underestimate the severity of drought conditions on the Korean Peninsula during the MM due to the limited constraints from historical rainfall data. This implies the presence of an amplification mechanism that enhanced the regulation of regional surface climate by solar cycle activity during the MM, which involves interactive ozone in the stratosphere, air–sea interaction processes, and the galactic cosmic rays (GCRs) affecting the troposphere via their forming condensation nuclei for clouds [2,3]. Some evidence appears to support the potential influence of GCR in our study region. Historical records of overcast days in Seoul over the past 400 years indicate a peak in persistent overcast conditions during the MM, suggesting increased low-altitude cloud cover in the region (Fig. S10). In the pre-industrial era, beyond the role of volcanic aerosols in promoting cloud formation, cosmic ray-induced ionization (CRII) may have been a significant factor in increasing low-altitude cloud cover [34]. Using the CRAC: CRII (Cosmic Ray Atmospheric Cascade: Cosmic Ray Induced Ionization) model [35], we calculated historical CRII at 3 km altitude over Seoul and found a significant increase in CRII during the MM (Fig. S10). Thus, a plausible explanation for the enhanced solar influence on Seoul's rainfall during the MM is that extremely low solar activity weakened the solar magnetic shielding, allowing more galactic cosmic rays (GCRs) to penetrate the Earth's atmosphere. These cosmic rays enhanced the formation of cloud condensation nuclei (CCN) through ion-induced nucleation, potentially increasing low-altitude cloud cover. The weakened solar radiation during the MM reduced surface and sea surface temperatures. The potential cloud-forming effect of cosmic rays further reduced the amount of solar radiation reaching the surface, amplifying the regulatory effect of solar activity on regional climate. Due to the difference in heat capacity between land and ocean, land cooling was more pronounced, leading to a weakened land-sea thermal contrast and a diminished EASM. The reduced water vapor transported by the EASM directly contributed to the prolonged drought in the interior of the Korean Peninsula.

**Figure 4.**
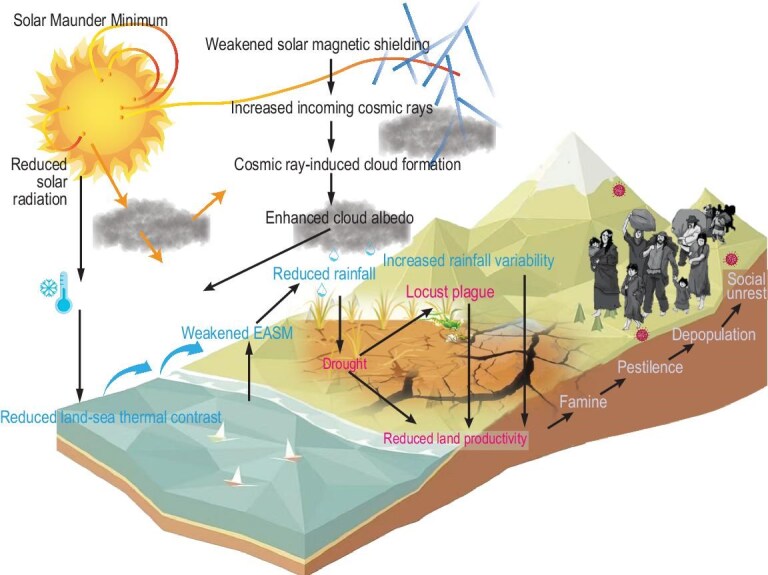
Conceptual model of regional climate changes induced by solar activity during the Maunder Minimum and the resulting series of ecological and societal crises.

Including but not limited to the aforementioned potential amplification mechanisms, which enhance the coupling between solar forcing and regional hydroclimate, these mechanisms exacerbated regional climate extremes and instability during the Maunder Minimum. These climatic conditions provide a compelling explanation for the social crises observed during the late Joseon Dynasty, including locust plagues, declining land productivity, crop failures, famine, pestilence, and short-term population declines. Finally, we integrate the evidence and discussions from this study into a conceptual model (Fig. 4). This model outlines a potential solar-climate-civilization cascade framework, offering a quantified historical perspective on the complex interactions between natural systems and human societies.

## DISCUSSION

The Joseon Dynasty's decline resulted from the combined effects of internal and external factors. After the devastating Imjin War (1592–1598 CE), the dynasty underwent reconstruction from the 17th–19th centuries. Late 17th-century political turmoil intensified factional struggles, causing frequent power transitions (‘hwanguk’). These conflicts disrupted governance while agricultural reforms and anti-Qing rhetoric increased peasant burdens, contributing to social instability and famines [37]. High-resolution climate reconstructions for Seoul reveal extreme droughts and climate instability during this period. In the mid-to-late 17th century, agricultural manuals like *Nongga Jipseong* and *Saekgyeong* incorporated newly added seasonal and geographical knowledge to address climate impacts, reflecting adaptation strategies that demonstrate climate change's direct effects on agriculture and daily life [38]. However, internal political turmoil and external climate change were not the direct causes of the dynasty's fall—the immediate cause was Japanese aggression during the 1894–1895 CE war, with colonial rule ultimately leading to the dynasty's collapse. Therefore, this study serves as a warning about potential extreme climate and social crises during periods of extreme solar activity, without supporting climate determinism. Instead, we observe the resilience of closed agricultural societies in responding to extreme climate. However, prolonged adverse climate conditions, frequent pest outbreaks, and repeated cycles of famine and epidemic gradually eroded society's foundations, ultimately weakening the dynasty's internal vitality to such an extent that it could not withstand foreign invasions, leading to its inevitable collapse.

Both historical observations and ^14^C-reconstructed sunspot numbers [20] show that long-term solar activity declined rapidly from ∼1600, suggesting the broader Maunder Minimum may have begun earlier than the defined 1645. Indeed, China, occupying a larger East Asian region, also experienced abnormal climate change and dynastic transition during this period. Late Ming climate deterioration (cooling, droughts, frosts, and frequent dry spells) reduced per capita grain production by 20%–50% across most regions, triggering widespread food crises. The severe drought of 1627–1643 became a key catalyst for peasant uprisings that ultimately defeated imperial forces and ended the Ming Dynasty [10]. In distant Europe, famines occurred repeatedly throughout the 17th century, with more recorded famine years than any other century [39]. Major famines struck in 1601–1602, 1623, 1648–1652, 1661–1662, 1693–1695, and 1697–1699, typically caused by combined climate shocks and warfare [11]. This demonstrates that extreme climate-induced agricultural crises around the Maunder Minimum were widespread across pre-industrial Eastern and Western civilizations, though specific climate characteristics varied regionally due to different geographical environments and climate circulation mechanisms.

When studying solar-climate relationships, we must acknowledge the field's complexity. On decadal timescales, solar activity's climate influence may be too subtle for direct observation, while the underlying physical mechanisms remain unclear. Thus, current observational and modeling studies have not definitively attributed certain anomalous climate changes to solar activity. Our rainfall reconstruction reveals extreme drought during the MM and decadal rainfall variability significantly correlated with sunspot cycles. Unlike previous studies, we introduce cosmic ray-induced cloud formation theory through observational data and the CRAC: CRII model to explain potential physical mechanisms of solar-climate influence, providing an important foundation for future exploration of solar-climate causality. Notably, although the Dalton Minimum (1790–1830 CE) was also a significant solar minimum with substantial climate and social changes (Fig. 3), volcanic eruptions are typically considered the primary external climate driver during this period [30], creating excessive interference for analyzing solar activity impacts.

The Maunder Minimum represents weakened solar magnetic activity, primarily controlled by the Sun's internal convection-driven dynamos [40]. Solar magnetic activity exhibits rich long-term variations, including not only grand minima but also grand maxima. For instance, the 20th century's Modern Grand Maximum, with its significantly enhanced solar radiation, largely contributed to global warming before 1970 [41]. Now, we shift our focus from historical periods to recent decades. Since the industrial era, anthropogenic forcing including carbon emissions has greatly exceeded natural forcing in observable climate impacts, and current global warming concerns are primarily driven by increased greenhouse gas emissions. However, in recent decades, overall solar activity has declined significantly. Solar Cycle 24 (2008–2019) exhibited sunspot and flare activity levels far lower than any previous cycles in the Space Age [42]. This decline has sparked discussions on whether the Sun might enter a ‘grand minimum’ phase. Our research raises a critical question: under current global warming conditions, what impacts would another Maunder Minimum have on future climate patterns and increasingly intelligent society? Atmospheric circulation model HadGEM2-CC simulations show that future weakened solar activity would enhance relative winter cooling over northern Eurasia and the eastern United States, reducing winter precipitation in northern Europe, partially mitigating but not offsetting overall global warming trends [43]. Our historical climate analysis for East Asia indicates that under severely weakened solar activity, cloud cooling effects strengthen along East Asian coasts, hydrological cycles weaken in inland East Asia, and solar cycle climate regulation significantly intensifies. Although modern society has undergone substantial changes in production and distribution compared to historical periods, ecological vulnerability and agricultural crises from climate change remain enormous challenges for societal development [44]. This means that comprehensively considering the combined responses of extreme solar activity, sunspot cycles [45], and greenhouse gas increases in future climate prediction models will serve as a crucial key to understanding future climate scenarios and societal dynamics.

## METHODS

Detailed descriptions of methods are available in the Supplementary Data.

## Supplementary Material

nwaf283_Supplemental_File
