# Supplementary material for: Cascading impacts of the Maunder Minimum on rainfall and society in the Joseon dynasty
Source: Natl Sci Rev. 2025 Jul 12;12(10):nwaf283. doi: 10.1093/nsr/nwaf283 (PMC12448634; doi:10.1093/nsr/nwaf283)
Supplement: nwaf283_Supplemental_File [file nwaf283_supplemental_file.pdf]

Supplementary Data for

Cascading impacts of the Maunder Minimum on rainfall and society in the Joseon dynasty

Yuqi Wang[1], Yong Wei[1,2*], Feng Shi[3], Xichen Li[4,5], Zhonghua Yao[6], Limei Yan[1,2], Yaochen Yue[1,2], Shiling Yang[2,3], Wei Lin[1,2], Yongxin Pan[1,2], Zhengtang Guo[2,3]

[1]Key Laboratory of Planetary Science and Frontier Technology, Institute of Geology and Geophysics, Chinese Academy of Sciences, Beijing, China;

[2]College of Earth and Planetary Sciences, University of Chinese Academy of Sciences, Beijing, China;

[3]State Key Laboratory of Lithospheric and Environmental Coevolution, Institute of Geology and Geophysics, Chinese Academy of Sciences, Beijing, China;

[4]Institute of Atmospheric Physics, Chinese Academy of Sciences, Beijing, China;

[5]Institute of Oceanography, Peking University, Beijing, China;

[6]Department of Earth Sciences, The University of Hong Kong, Hong Kong, China

Correspondence: Yong Wei

Email: weiy@mail.iggcas.ac.cn

This PDF file includes:

Methods
Figs. S1 to S16
Tables S1 to S6
Note S1

METHODS

Rainfall data quality assessment

Over the past 400 years, four high-resolution, continuous rainfall datasets have been available for the Seoul region: Ancient Rainy Days (ARD, 1623–1910 CE) [13,14], Ancient Rainfall Amounts (ARA, 1778–1907 CE) [13,15], Modern Rainy Days (MRD, 1908 CE–present), and Modern Rainfall Amounts (MRA, 1908 CE–present). Modern rainfall data (post-1908 CE) are derived from the climate statistics of the Korea Meteorological Administration's National Climate Data Center. High-precision rainfall records (mm/2 hours) from 1778 to 1907 CE, measured using ancient rain gauges, have been well-preserved and utilized in long-term hydrometeorological analyses for Seoul [12].

Compiling climate records from historical documents to reconstruct long-term climate variability is a widely used approach in historical climate research for East Asia. Given the complexity and fragility of historical records, evaluating their reliability and continuity is a critical step to ensure the accuracy of reconstruction results. The rainfall records used in this study are derived from the *Seungjeongweon Ilgi* (The Daily Records of the Royal Secretariat of the Joseon Dynasty), which is recognized as the largest chronological document in the world. The surviving Seungjeongweon Ilgi provides detailed documentation of 288 years of political, agricultural, meteorological, and other begins with a record of the day's weather conditions (**Fig. S11**), followed by descriptions of the king's public activities, interactions with bureaucratic institutions, royal decrees, official documents, and societal events. Remarkably, even the king himself was prohibited from accessing these records, ensuring the authenticity of the entries.

The weather data from the *Seungjeongweon Ilgi* have been systematically compiled and evaluated, demonstrating promising applications in climate research. The compilation of the Seungjeongweon Ilgi was primarily the responsibility of the officials of the State Council, with a daily record maintained by one official and two Hanlin scholars who accompanied the king. They documented current events by referring to documents handled by the State Council as well as weather conditions, transcribing these into daily diaries, which were compiled into volumes at the end of each month. This diary format ensured high temporal resolution and homogeneity of the original records. Due to wars, fires, and other factors, the surviving Seungjeongweon Ilgi spans from March of the first year of King Injo's reign (1623) to August of the fourth year of King Gojong's reign (1910), comprising a total of 3,244 volumes, which are preserved at the Kyujanggak Library of Seoul National University. Despite efforts to restore the records after the historical destruction caused by wars and fires, there remains a certain degree of missing weather data. We analyzed all weather records (e.g., sunny, cloudy, rainy, snowy, etc.) from the Seungjeongweon Ilgi between 1625 and 1910 CE and found that, on average, more than 340 daily weather entries were recorded annually. Most missing records are concentrated after 1900 due to the impacts of foreign invasions (**Fig. S12**). Only ten years have fewer than 300 weather records (1695, 1730, 1772, 1852, 1894, and 1900–1904 CE), with the majority of these gaps attributable to wars and fires (**Table S2**). From the perspective of annual rainfall reconstruction, the continuity of these data is equivalent to near-uniform sampling, ensuring the reliability of the historical dataset.

To promote stable agricultural production, King Sejong and a group of scholars invented the Chukwookee (a rain gauge), which standardized rainfall measurements to an accuracy of millimeters per two hours. However, due to significant wars and fires, early rainfall observation records were severely disrupted. In 1770 CE, King Yeongjo restored the rainfall observation system, resuming daily rainfall measurements at the royal palace in Seoul. These measurements continued for approximately 130 years until 1907, when the Chukwookee was replaced by modern rain gauges. Today, the reconstructed Ancient Rainfall Archive (ARA) for Seoul from 1778 to 1907 CE provides a complete time series after addressing missing data and reducing errors from manual readings. This dataset represents a critical resource for studying historical rainfall in Seoul [15].

Reconstruction of rainfall using Generalized Additive Model

Generalized Additive Model (GAM) is a flexible extension of generalized linear model (GLM) that allow for the modeling of non-linear relationships between predictors and a response variable by using smooth functions [46]. Unlike traditional linear models, which assume a fixed linear relationship between variables, GAM use smoothing splines or other basis functions to capture complex, non-linear patterns in the data. This flexibility makes GAM particularly suitable for environmental and climate studies, where relationships between variables are often non-linear and influenced by multiple interacting factors. The general form of a GAM is expressed as:

$$g(\mu) = \beta_0 + f_1(x_1) + f_2(x_2) + \dots + f_p(x_p)$$

Where $g(\mu)$ is the link function, β_0 is the intercept, and $f_i(x_i)$represents smooth functions of the predictor variables x_i . These smooth functions are estimated from the data using methods such as cubic regression splines or thin-plate splines, with penalties applied to avoid overfitting. The degree of smoothness is typically determined by cross-validation or generalized cross-validation (GCV).

In this study, the response variable of the model is the annual/summer rainfall from 1780 to 2000 CE, which includes ancient observed rainfall data prior to 1908 CE and modern meteorological observations after 1907 CE. Between 1780 CE and 1907 CE, there were a total of 7,916 ancient observational rainfall records, and **Fig. S13** illustrates the statistical distribution of these data. Over 70% of the total rainfall occurred between June and September (**Fig. S13a**), with daily rainfall peaks typically occurring between 3 AM and 6 AM (**Fig. S13b**). In this study, we focus on the long-term variations in rainfall in Seoul, therefore, we summed all rainfall records for each year to obtain the total annual rainfall, denoted as "annual rainfall (mm)." Similarly, we summed all rainfall records from June to August to calculate the total summer rainfall for that year, denoted as "summer rainfall (mm)." The predictor variables include annual rainfall days from 1780 to 2000 CE, Northern Hemisphere mean temperature (NT), the Pacific Decadal Oscillation (PDO) , El Niño–Southern

Oscillation (ENSO), North Atlantic Oscillation (NAO) , Atlantic Multidecadal Oscillation (AMO) and the Sunspot Number (SN).

The model fitting results reveal that annual rainfall days and NT exert significant influences on the reconstruction of annual rainfall, with 54% of the variability in annual rainfall fully explained by changes in rainfall days (**Table S4**). To detect the sensitivity of the regression model to the selection of climate variable datasets, we tested multiple reconstructed datasets of the same variable (**Table S3, S4**). Based on the significance of the model fits for each variable (p-values), there is considerable variability in the sensitivity of the fitting results to different NT datasets, while the sensitivity to other climate variable datasets is relatively low. This may be attributed to the fact that our reconstructed rainfall data is inherently sensitive to changes in NT, leading to significant differences in responses to various temperature datasets. Consequently, we ultimately selected datasets with a reconstruction significance of $p < 0.05$ as model variables. To evaluate the performance of all possible combinations of predictor variables, we employed the Generalized Cross-Validation (GCV) score. GCV assesses a model's explanatory power by minimizing the generalized estimate of prediction error while imposing a penalty on overly complex models (i.e., those with excessive smoothing parameters or degrees of freedom). A lower GCV score indicates that the model effectively captures data features while avoiding overfitting, thereby achieving better generalization. Among all possible predictor variable combinations, the model with the lowest GCV score included rainfall days, NT, and PDO (**Table S5**). Consequently, these three variables were selected as predictors for constructing the regression model for annual rainfall. Notably, the deviance explained by the model is 60%, indicating that it accounts for 60% of the observed variability in annual rainfall.

For example, to calculate the annual rainfall for the year 1650, we first used the 128 annual rainfall records from 1780 to 1907 as the response variable, along with annual rainfall days, NT, and PDO from the same period as predictor variables in a Generalized Additive Model (GAM). This allowed us to obtain a smooth function that characterizes the relationship between the response and predictor variables. We employed the mgcv package in R with an identity link function to represent this relationship (**Fig. S14**). Next, we input the values of annual rainfall days, NT, and PDO for 1650 as predictor variables into the GAM model, which then calculated the annual rainfall for that year based on the learned function. The calculation for summer rainfall each year follows the same approach. It is important to emphasize that this study can only reconstruct annual rainfall and summer rainfall, and cannot be used to reconstruct monthly resolution rainfall. Since rainfall in Seoul is primarily concentrated in the summer, there is sufficient data available for model construction during this period (as verified by residual testing), whereas rainfall in other months is too sparse, making the reconstruction results for those months unable to pass residual tests, thus making it unsuitable for separate reconstruction of other months.

Reconstruction validation

To validate the reconstructed rainfall model, we used the DHARMa package in R, which provides a comprehensive framework for residual diagnostics in regression models. Residual

diagnostics are essential for assessing the validity of the model assumptions and the quality of the fit.

The DHARMa package simulates residuals from the fitted model to create a uniform distribution under the null hypothesis that the model is correctly specified. These simulated residuals are independent of the original data and can be used to test for deviations from model assumptions, such as heteroscedasticity, autocorrelation, or non-normality. Key diagnostic tools used in this study include QQ Plot of Residuals and Residuals vs. Predicted Plot. The QQ plot compares the empirical distribution of the simulated residuals to the expected uniform distribution. Deviations from the diagonal line indicate potential violations of the model assumptions, such as skewness or heavy tails in the residuals. In our study, the QQ plot showed a good alignment with the uniform distribution, indicating no significant deviations from the model assumptions. Residuals vs. Predicted Plot examines the relationship between residuals and predicted values. A random scatter of points around zero suggests that the model has captured the underlying patterns in the data without systematic bias. In our results, the residuals vs. predicted plot revealed no discernible patterns, confirming that the model adequately captured the non-linear relationships between rainfall and the predictors (**Fig. S15**). In order to further evaluate the overall fitting effect of the model, statistical fidelity test was conducted on the model. The reconstruction demonstrates positive skill, with the average reduction of error (RE) are 0.43 and 0.48 and the average coefficient of efficiency (CE) are 0.39 and 0.45 for the two verification periods, indicating agreement between actual and estimated rainfall data exceeding climatology (**Note. S1**).

Social response index

The economy of the Joseon Dynasty experienced stagnation and decline during the 18th and 19th centuries. Evidence suggests that the continuous decline in agricultural productivity, particularly in paddy field productivity, was one of the key factors contributing to the economic contraction in late Joseon [32]. Paddy field productivity can be quantified through paddy field rents. In general, paddy field rents are proportional to land productivity, and a decline in rents reflects a decrease in land productivity. From 1685 to 1945 CE, the long-term trend of paddy field rents per unit area (measured in doh) in the Joseon Dynasty showed a persistent decline [36]. This decline may have been driven by population expansion during this period, which caused a surge in demand for timber, leading to widespread deforestation. The resulting soil erosion and land degradation further reduced agricultural productivity. The ecological and socio-environmental changes caused by forest degradation are typically slow processes that manifest on centennial timescales [47]. To focus on the impact of climate variability on paddy field productivity at decadal timescales, we removed the long-term linear trend from the paddy field rents. The detrended variations in paddy field rents better reflect the influence of climate variability on productivity at shorter timescales (**Fig. S16**). Thus, in this study, we use detrended paddy field rents as a proxy for land productivity (**Fig. 3E**).

The population index for the late Joseon Dynasty used in this study was estimated based on the dynastic Household Survey [33]. This series is approximately 2.5 times higher than the official population records, as the actual resident population likely exceeded the officially

registered population due to the presence of transient individuals. A key purpose of population registration in the Joseon Dynasty was to impose taxes on the lower classes, meaning that the vast majority of the recorded population were farmers who were most vulnerable to famine. In contrast, the population of resident literati and more superior royal families was recorded separately [48]. These groups were excluded from our analysis because they were less directly affected by agricultural production and famine.

The locust, famine, and pestilence indices used in this study were derived from reports submitted by local officials to the king, which were recorded in the Seungjeongweon Ilgi (https://sjw.history.go.kr/main.do). We systematically extracted all reports related to locust outbreaks, famines, and epidemics from the *Seungjeongweon Ilgi*, each of which contains precise dates down to the specific day. However, due to information transmission delays and other logistical factors, these events were often reported to the royal government days to months after their actual occurrence. Therefore, we summed the annual locust (famine/ pestilence) events recorded from 1625 to 1900 to obtain an annual index of these disaster events, representing the number of occurrences each year. This approach provides a more reasonable representation of the cumulative disaster burden experienced in each year. It is important to note that not all disaster events that actually occurred were comprehensively reported, as events with broader geographic impact or more severe societal consequences were more likely to be documented and submitted to the royal government. Therefore, these indices represent a composite measure of both the frequency of these events and their societal impact, rather than simply counting isolated occurrences.

Figs. S1 to S16

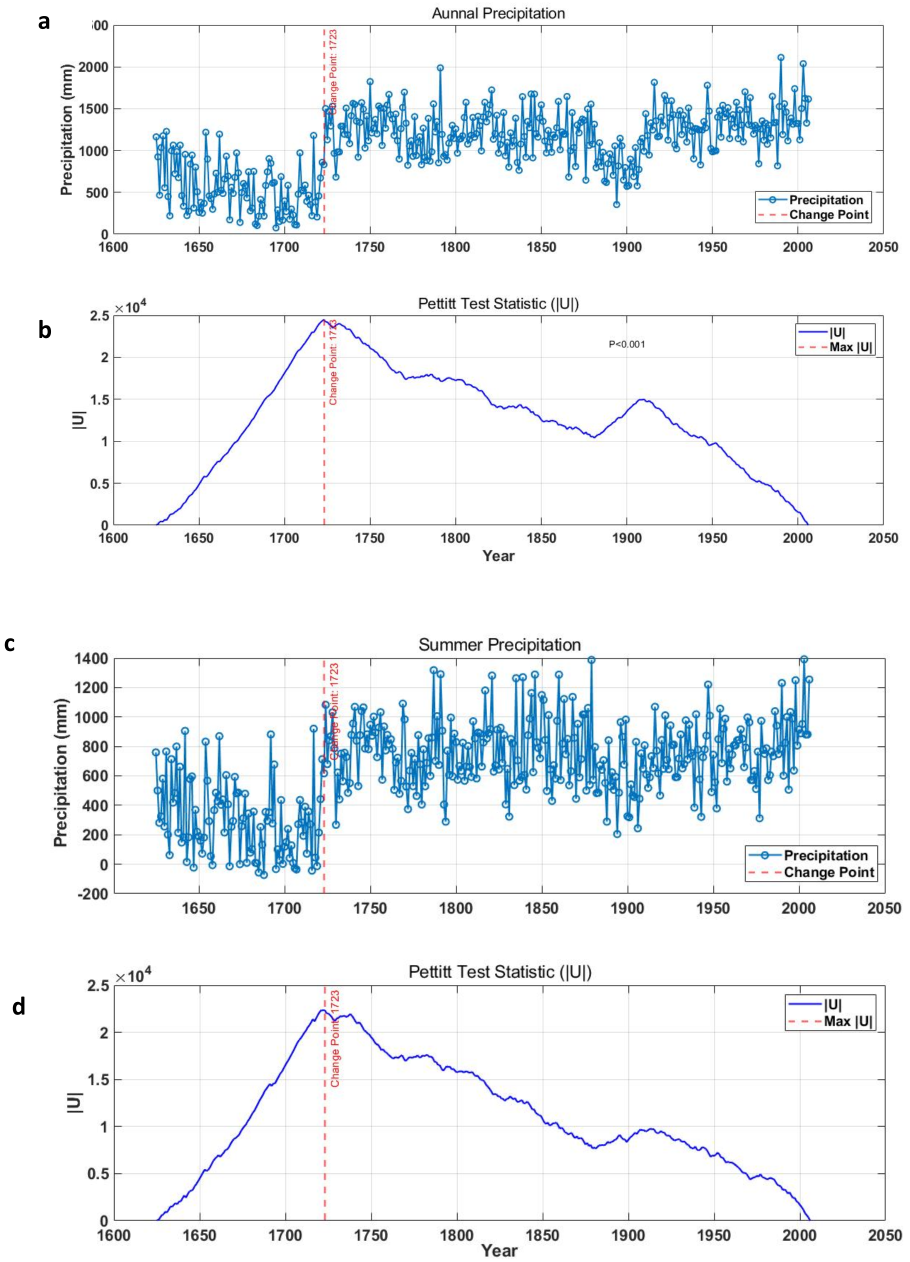

Figure S1. Petitt test of abrupt change point of annual rainfall in Seoul. a, time and mutation point of annual precipitation. b, the fluctuation of the Petitt test statistic, the largest fluctuation in 1723. c and d, the same as a and b, nut for summer.

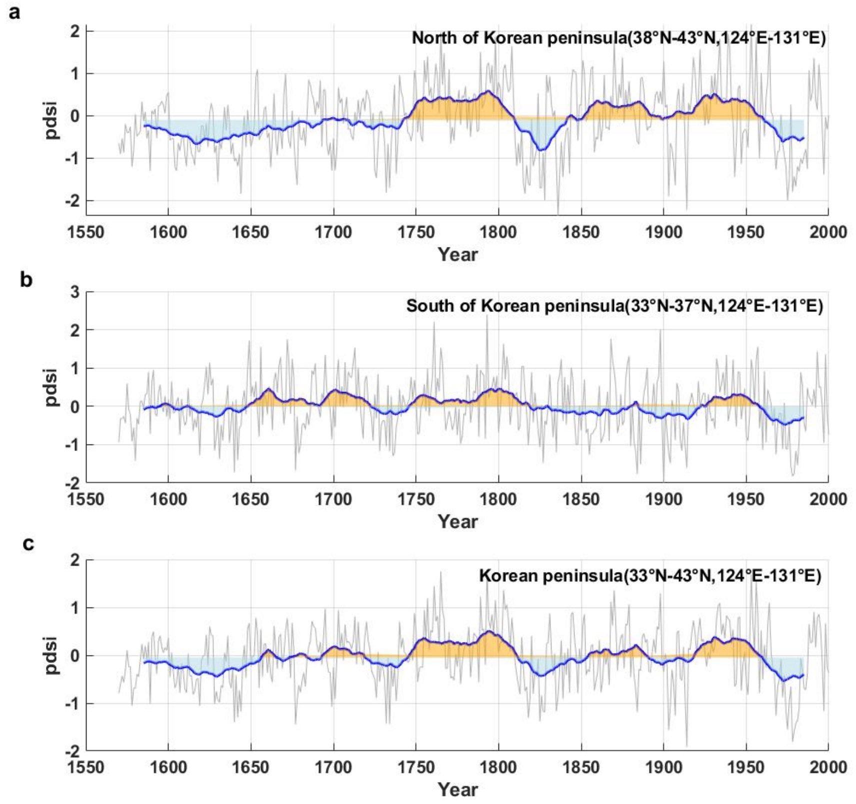

Figure S2. Summer (June to August) Palmer Drought Severity Index (PDSI) reconstructed by Palaeohydrodynamic data assimilation products (PHYDA) in Korean Peninsula [18]. The parts above the mean are filled in yellow, and the parts below the mean are filled in blue. a, North of Korean peninsula(38°N-43°N,124°E-131°E). b, South of Korean peninsula(33°N-37°N,124°E-131°E). c, Korean peninsula(33°N-43°N,124°E-131°E).

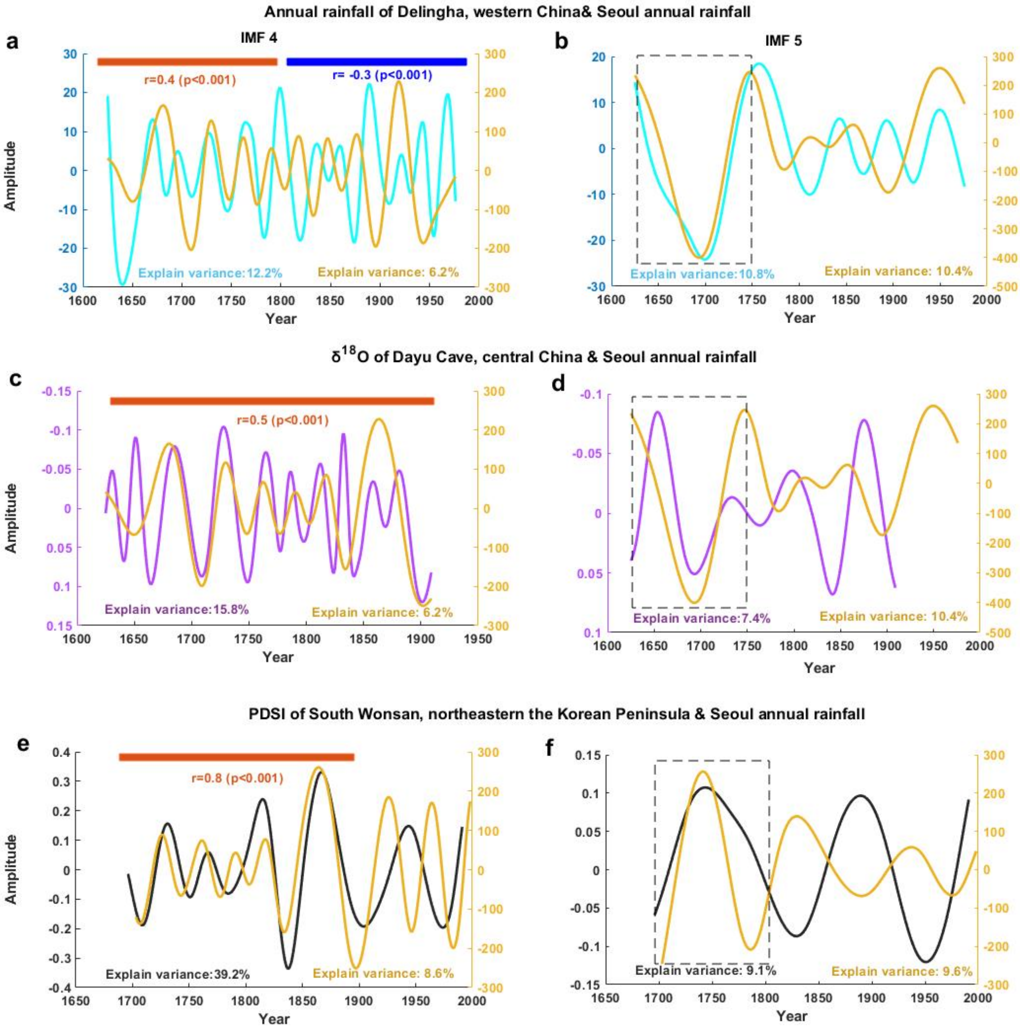

Figure S3. Empirical modal decomposition of rainfall data. The yellow curve shows the interdecadal and multidecadal change represented by the different IMF components of Seoul's annual rainfall. The blue curve shows the IMF weight of tree-ring-based rainfall reconstruction in the Delingha region of Qinghai. The purple curve represents the IMF component of speleothem $\delta^{18}O$ rainfall reconstruction at Dayu Cave near the Qinling Mountains. The black curve represents the IMF component of pdsi index reconstructed from tree-ring data in South Wonsan (38.1°N, 128.4°E) in northeastern Korea. Refer to Fig. 1a for specific point distribution.

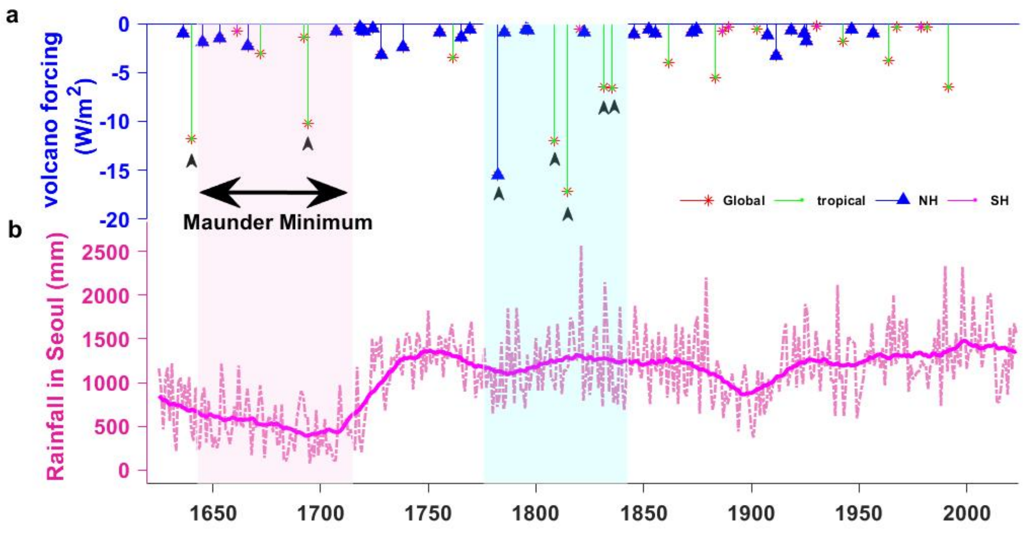

Figure S4. Global volcanic eruption sequence. a, Radiative forcing caused by different types of volcanic aerosols [27]. Tropical volcanoes are shown in green, northern Hemisphere volcanoes in blue and southern Hemisphere volcanoes in pink. b, Reconstructed annual rainfall in Seoul and 30-year sliding average (yellow curve and orange bold curve, respectively).

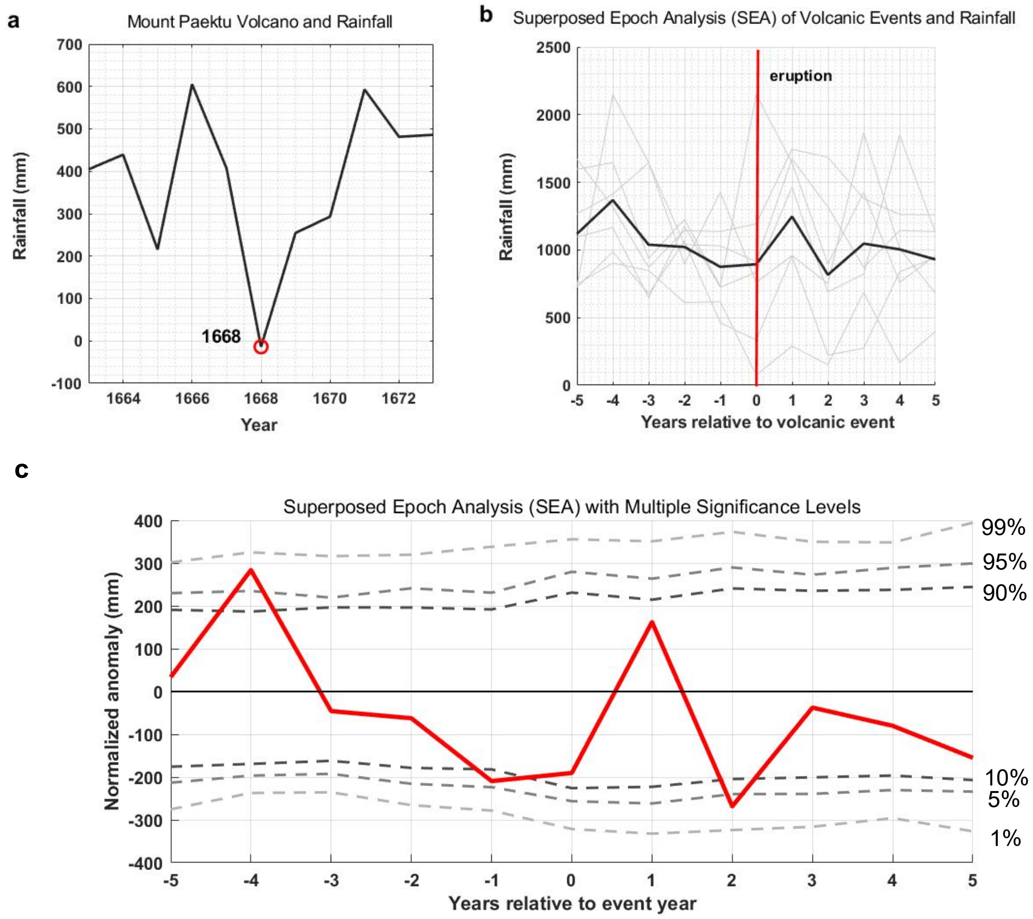

Figure S5. Response of rainfall in Seoul to volcanic activity. a, Summer rainfall variation between 1663 and 1673 CE. In 1668 CE, the volcanic eruption of Mount Baekdu on the Sino-Korean border coincided with the lowest summer precipitation in Seoul. b, Superposed Epoch Analysis of Seoul rainfall response to seven global intense volcanic events (as indicated by the arrows in Figure S4a, they erupted in 1641, 1695, 1783, 1809, 1815, 1832 and 1836 CE, respectively). The grey line in the background shows the rainfall series in Seoul for a total of 10 years before and after each volcanic eruption, and the thick black line is the average of all rainfall series. (c) Significance testing of SEA for the impact of volcanic eruptions on rainfall anomalies. The red line represents the mean rainfall response averaged across all volcanic events, while the dashed gray lines indicate the 90%, 95%, and 99% confidence intervals calculated using 1,000 random resampling iterations. The darkest gray dashed lines represent the 90% significance bounds, the medium gray dashed lines represent the 95% significance bounds, and the lightest gray dashed lines represent the 99% significance bounds. Year 0 marks the volcanic eruption year, with a time window spanning from 5 years before to 5 years after the event. If the red line (observed response) exceeds the corresponding dashed lines, it indicates that the response for that year is significant at the respective significance level (90%, 95%, or 99%). The results show no significant impact of volcanic eruptions on rainfall in Seoul.

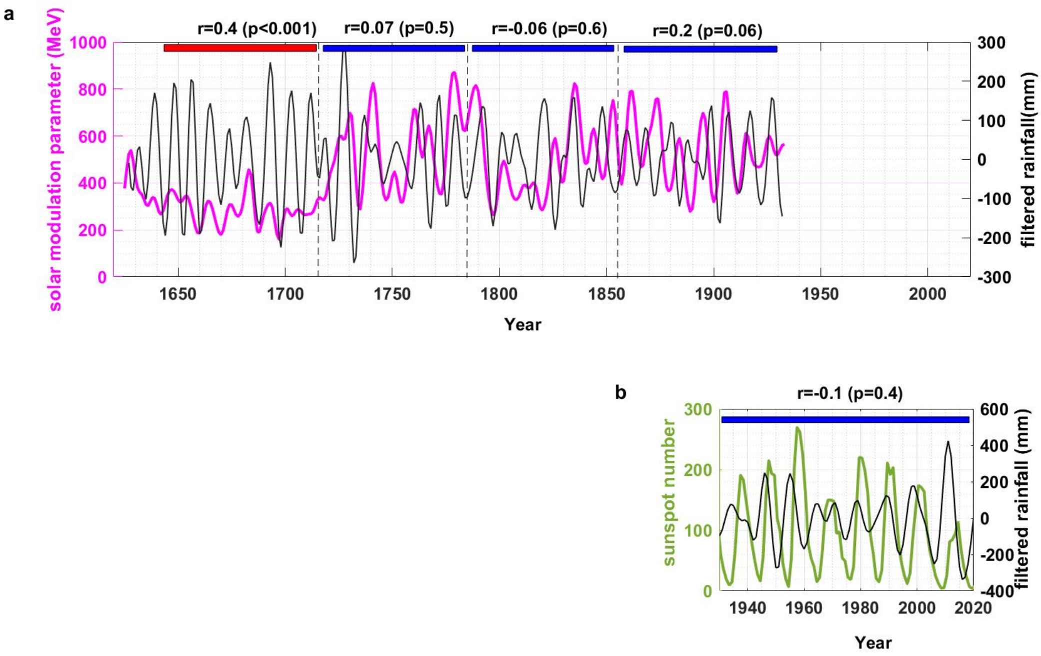

Figure S6. Interdecadal correlation between Seoul rainfall and solar activity from 1625-2023 CE. a, Time series of solar modulation potential and Seoul rainfall after 6-15 year bandpass filtering. Colored bands represent correlations within approximately 70-year intervals following the onset of the Maunder Minimum (1645-1715 CE), with red indicating periods of significant correlation and blue indicating periods of non-significant correlation. b, Bandpass-filtered modern observed sunspot numbers and Seoul annual rainfall.

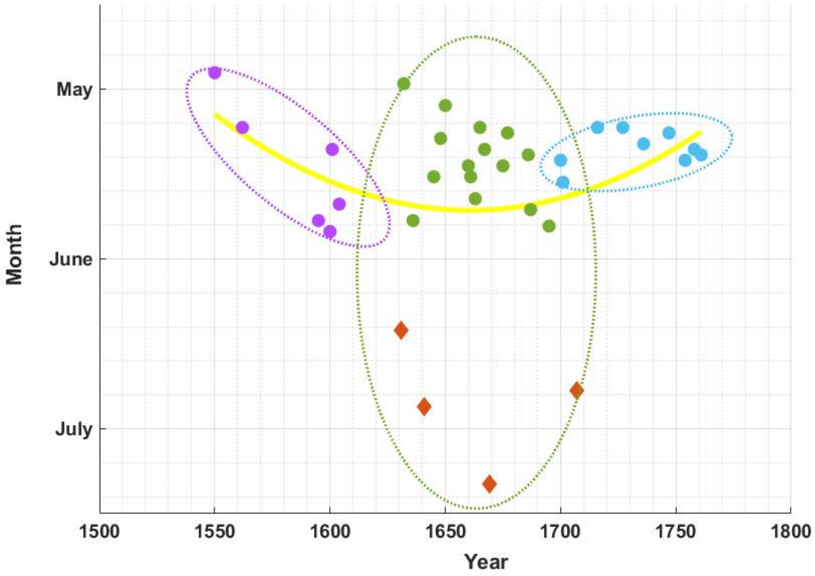

Figure S7. The time of the late frost (the last frost before July 30 each year) event of the Joseon Dynasty. Frost events are divided into four categories, each marked by a different color. The 17th century saw the latest frosts [31].

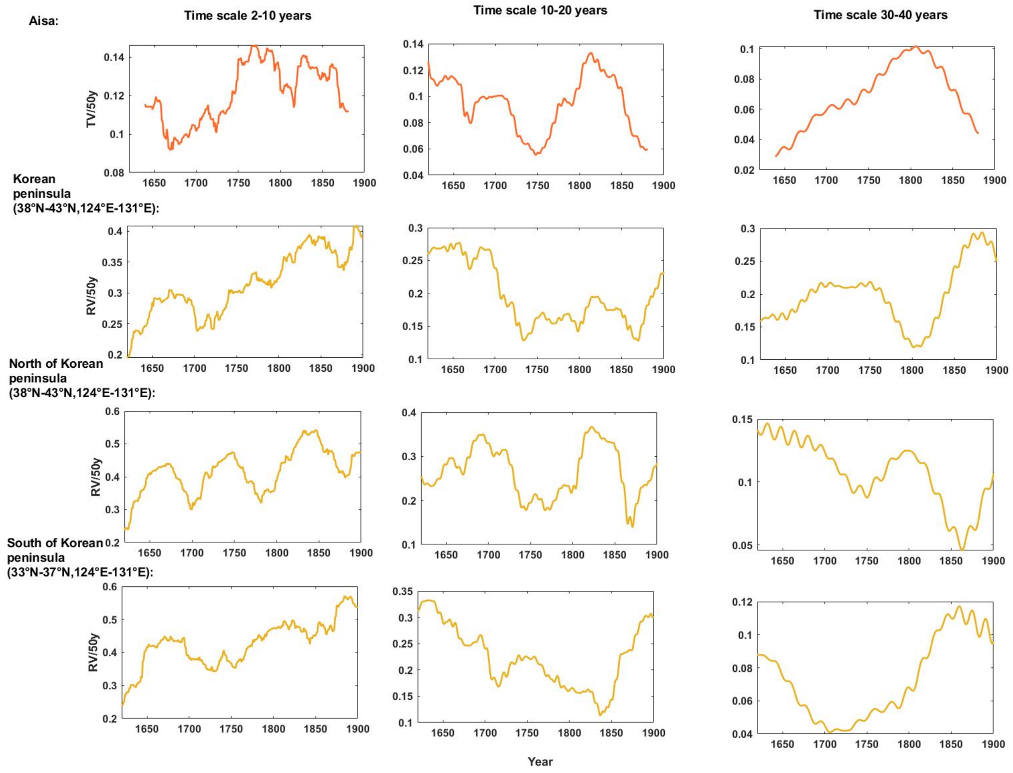

Figure S8. Rainfall/temperature variability under different time scales (RV/TV is defined as the standard deviation of rainfall/temperature after removing the 100-year long-term trend, the standard deviation is calculated with a 50-year window and 1 year as the sliding step length).

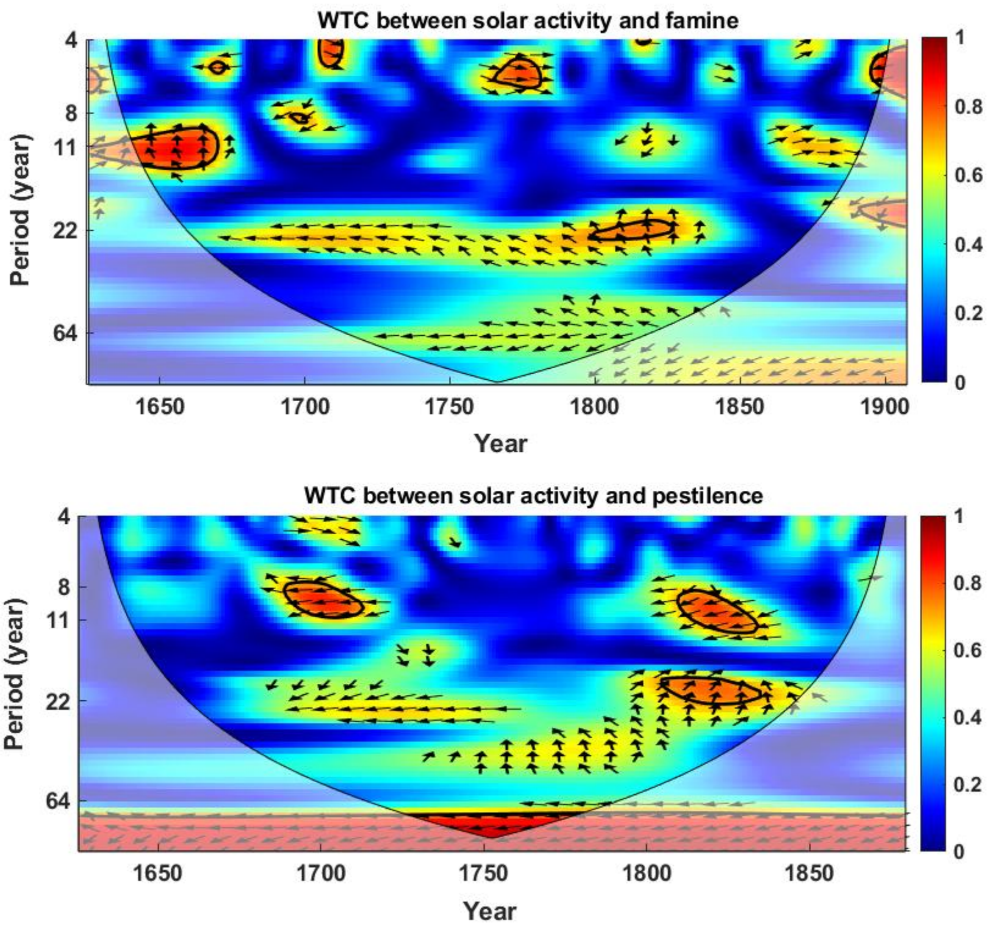

Figure S9. Wavelet coherence analysis (CWT) of late Li Dynasty famine/plague and solar activity (expressed as solar modulation potential). The arrow length represents the coherence intensity of the two variables, the arrow direction represents the coherence phase of the two variables, and the right represents the complete positive coherence and the left represents the complete negative coherence. Bold black outline indicates 95% significance level.

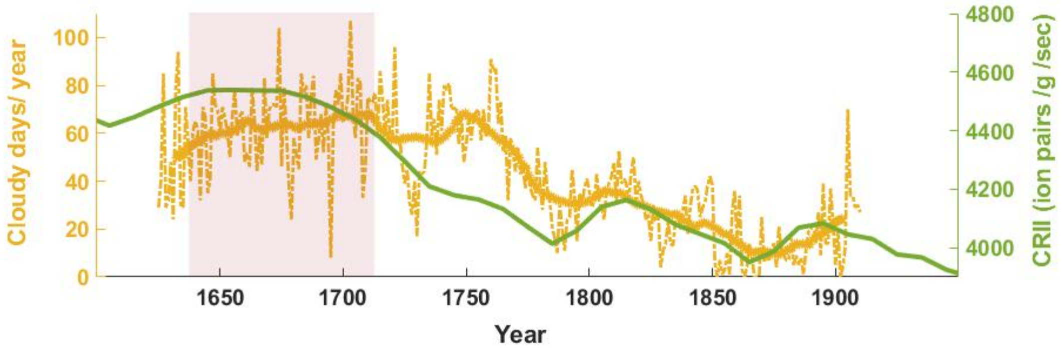

Figure S10. Annual change in cloud cover over Seoul (dotted yellow line) and its 30-year sliding average (solid yellow line). The solid green line represents the calculated Cosmic Ray Induced Ionization (CRII) using CRAC: CRII model, which represents the logarithm of ionization produced per cubic centimeter of air per second, and represents the contribution of cosmic rays to the ionization process in the Earth's atmosphere [35].

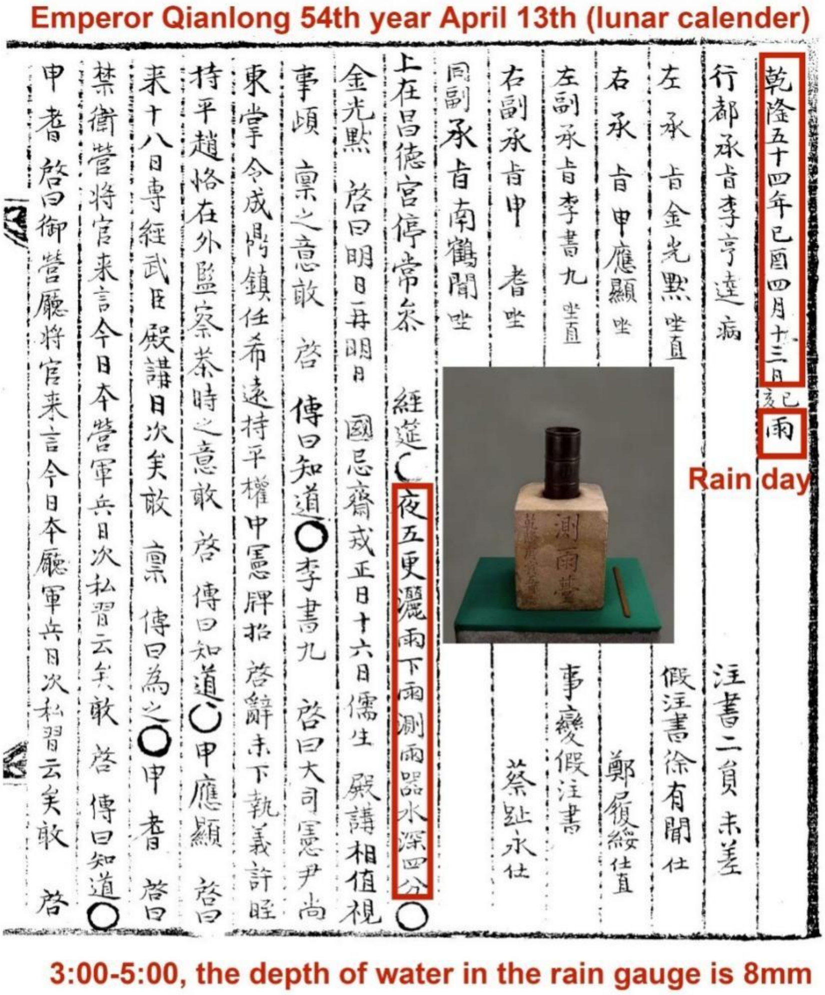

Figure S11. An example rainfall records in Seungjeongweon Ilgi, accompanied by a picture of the rain gauge. The scanned copy of this record can be accessed at the National Institute of Korean History (http://sjw.history.go.kr/id/ SJW-G13040130-00200).

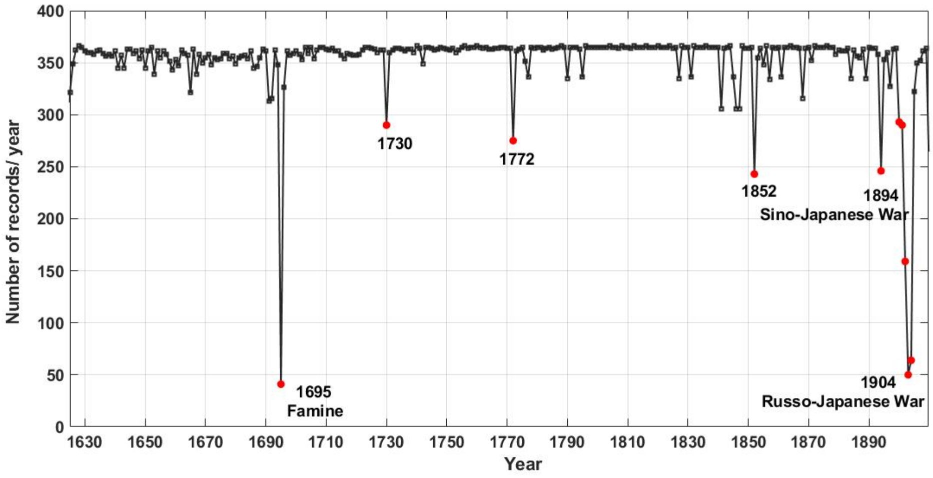

Figure S12. The number of annual weather records in Seungjeongweon Ilgi. Years with fewer than 300 weather records per year are indicated by red dots.

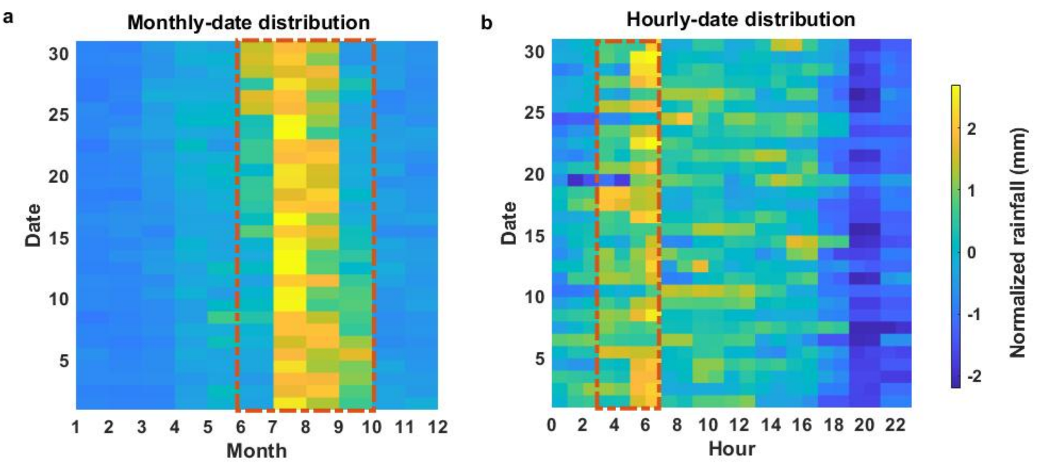

Figure S13. The temporal distribution of ancient rainfall from 1780 to 1907 CE. a, Monthly-Date distribution of rainfall. b, Hourly-Date distribution of rainfall. The color bar represents the normalized rainfall anomaly.

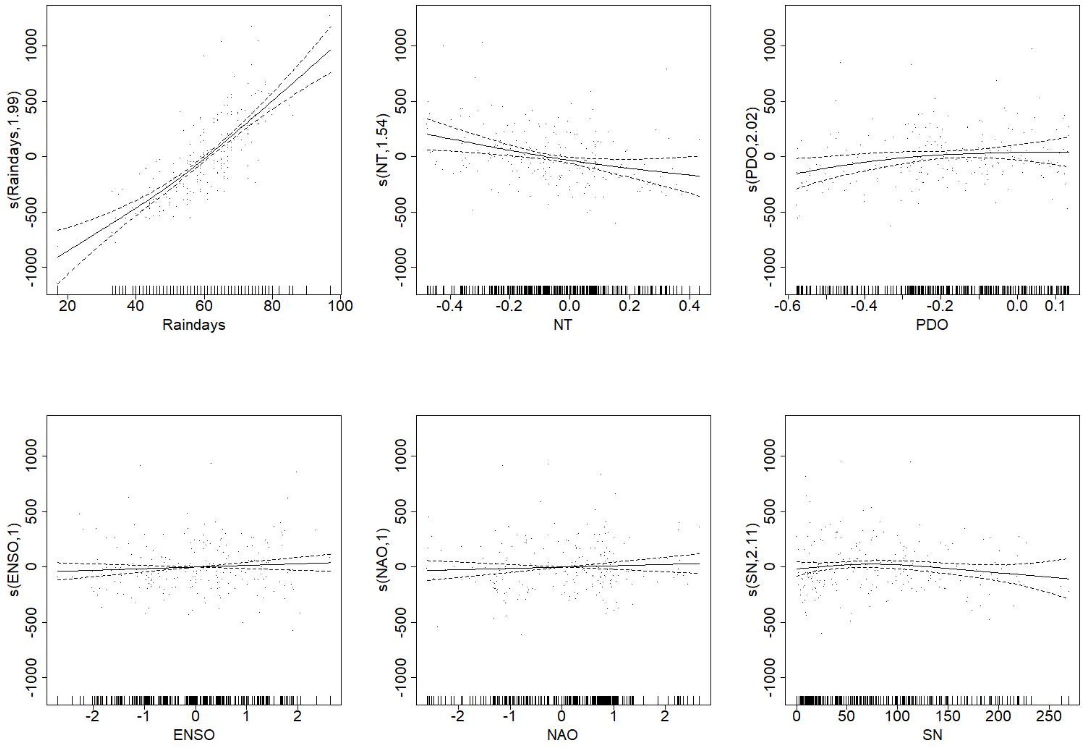

Figure S14. Partial effects of the smoothing terms of each predictor variables for GAM model diagnostics on rainfall. predictor variables include Raindays, Northern Hemisphere Mean Temperature (NT), Pacific Decadal Oscillation (PDO), and El Nino associated with the Southern Oscillation (ENSO) and the North Atlantic Oscillation (NAO) and the sunspots. The X-axis represents the value of the argument for the term that should be smoothed. The Y-axis represents the contribution of the smoothing term to the response variable (the effect of deviation from the overall mean), and the number in s(PDO, 1.92) represents the Effective Degrees of Freedom (EDF) of that smoothing term. The solid line is the estimated effect curve of the smooth term, and the dashed line is the 95% confidence interval. If the curve is close to a straight line, the effect of the smoothing term on the response variable is close to linear. If the curve has a significant bend, it indicates a nonlinear effect. The short line at the bottom shows where the data points are distributed.

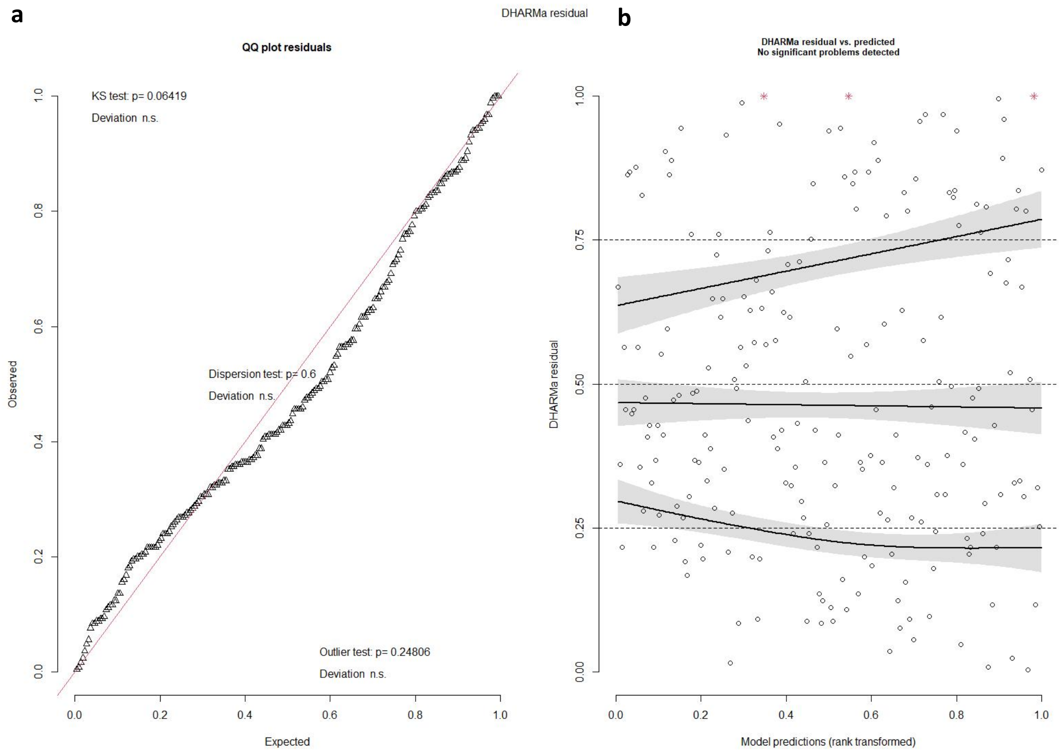

Figure S15. The residual test of GAM model. a, Residual QQ chart. It represents the difference between the actual distribution of the residual and the expected distribution of the normal distribution. The residual points are approximately evenly distributed on a diagonal line, indicating that the residual is normally distributed. b, The residual test shows no significant problems detected.

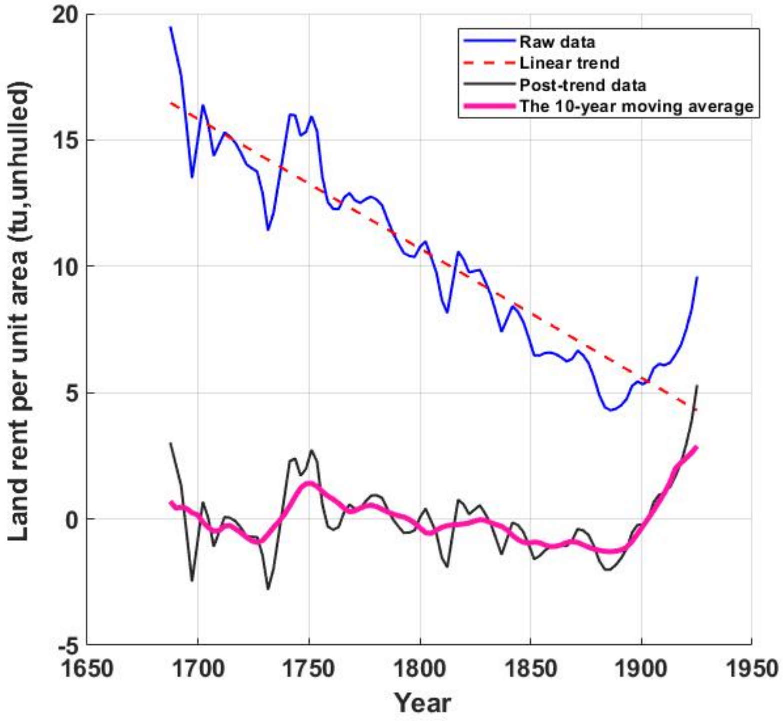

Figure S16. Joseon Dynasty paddy field rent per unit area (in dou, blue curve) and its long term linear trend (red dashed line). The solid black line is the paddy field rent after subtracting the long-term linear trend. The pink curve is the 30-year moving average of the black curve.

Tables S1 to S6

Table S1：Overview of the measured and reconstructed rainfall comparison. Values are ensemble medians and 2.5th and 97.5th percentiles are shown in square brackets.

Period	M/R variance ratios	M/R correlations
Annually	1.33 [1.14 1.78]	0.72 [0.63 0.88]
Summer	1.36 [1.10 1.68]	0.76 [0.53 0.80]

Tabel S2. A list of fewer than 300 weather records in a year in the Seungjeongweon Ilg

Year (CE)	Number of total weather records	Events
1695	41	fire
1730	290	NAN
1772	275	NAN
1852	243	fire
1894	246	the Sino-Japanese War， fire
1900	293	NAN
1901	290	NAN
1902	159	NAN
1903	50	NAN
1904	64	aggression war

NAN means no record.

Tabel S3. Predictor variables reconstructions used in this paper.

Index	Source
NT1	DOI: 10.1126/science.ll77303
NT2	DOI: 10.1038/nature03265
PDO1	DOI: 10.1038/nclimate1086
PDO2	DOI: 10.1029/2005GL022478
ENSO1	DOI: 10.1038/nclimate1086
ENSO2	DOI: 10.1175/1520-0442(2000)013<1517:PEATHD>2.0.CO;2
AMO1	DOI: 10.1038/ngeo2962
AMO2	DOI: 10.5194/cp-14-157-2018
NAO	DOI: 10.1126/science.1166349
SN	WDC-SILSO, Royal Observatory of Belgium, Brussels

Table S4： GAM model parameters. edf: The effective degrees of freedom for the smooth term, indicating the degree of smoothing. Higher values suggest stronger nonlinearity. Ref.df: The reference degrees of freedom. F: The F-test value for the smooth term. p-value: The significance level of the smooth term, indicating whether the variable has a significant effect on the response variable.

Formula: Rainfall ~ s(Raindays) + s(NT) + s(PDO) + s(ENSO) + s(NAO) + s(SN)				
Formula	edf	Ref.df r	F	p-value
s(Raindays)	2.126	2.737	79.597	< 2e-16***
s(NT1)	1.000	1.000	4.523	0.0348*
s(NT2)	2.855	3.623	0.927	0.4320
s(PDO1)	1.682	2.099	2.404	0.0819.
s(PDO2)	1.000	1.000	0.236	0.6274
s(ENSO1)	1.000	1.000	1.028	0.3119
s(ENSO2)	1.000	1.000	1.216	0.3227
s(AMO)	1.000	1.000	0.804	0.3710
s(AMV)	1.000	1.000	1.032	0.3110
s(NAO)	1.000	1.000	0.500	0.4801
s(SN)	1.000	2.633	1.485	0.3072

Signif. codes: 0 ‘***’ 0.001 ‘**’ 0.01 ‘*’ 0.05 ‘.’ 0.1

adjusted R-squared = 0.57 Deviance explained = 60.4%

Table S5： Generalized cross-validation (GCV) scores for different models. The smaller the GCV score, the better the fitting result of the model. Here are the top five models.

Model	Formula	GCV
Model 1	Rainfall ~ s(Raindays) + s(NT1) + s(PDO1)	60246.37
Model 2	Rainfall ~ s(Raindays) + s(NT1) + s(PDO1)	60352.04
Model 2	Rainfall ~ s(Raindays) + s(PDO1) + s(NAO) + s(AMO2)	60445.10
Model 3	Rainfall ~ s(Raindays) + s(SN) + s(PDO1) + s(AMO2) + s(NAO)	60049.64
Model 4	Rainfall ~ s(Raindays) + s(SN) + s(PDO2) + s(ENSO1) + s(AMO1)	61623.59
Model 5	Rainfall ~ s(Raindays) + s(NAO) + s(PDO2) + s(ENSO1) + s(AMO2)	61946.79
…	…	…
Model 32	Rainfall ~ s(Raindays)	66047.91
…	…	…

Table S6. Calibration and verification statistics for the period of 1780-2023 CE.

Calibration period	*r*	R^2	Verification period	RE	CE
Full period (1780-2023)	0.73	0.53			
Early half (1780-1900)	0.76	0.58	Late half (1901-2023)	0.43	0.39
Late half (1901-2023)	0.72	0.52	Early half (1780-1900)	0.48	0.45

Note S1：Statistical fidelity test

The whole period is divided into two equal sections, 1780-1900 CE and 1901-2023 CE, which take turns as the calibration period and verification period. Four statistical indicators are calculated to test the goodness of fit between the actual and estimated rainfall in each period. The Pearson correlation coefficient (r) is to 1, the stronger the correlation is. The coefficient of determination (R2) which indicates the degree of explanation of the predictor variables on the response variable is given as

$$R^2 = 1.0 - \frac{\sum(x_i - y_i)^2}{\sum(x_i - \bar{x})^2}$$

The average reduction of error (RE) is given as

$$RE = 1.0 - \frac{\sum(x_{iv} - y_{iv})^2}{\sum(x_{iv} - \bar{x}_c)^2}$$

where xi and yi are the actual and estimated rainfall data in year i of the full period, $\bar{x}$ and $\bar{y}$ are the means of the actual and reconstructed rainfall data respectively of the full period, xiv and yiv are the actual and estimated rainfall data in year i of the verification period and $\bar{x}_c$ is the mean of the actual data in the calibration period. The average coefficient of efficiency (CE) is given as

$$CE = 1.0 - \frac{\sum(x_{iv} - y_{iv})^2}{\sum(x_{iv} - \bar{x}_v)^2}$$

where $\bar{x}_v$ is the mean of the actual data in the verification period. Both RE and CE have a theoretical range between -∞ and +1. If their values are 1, the agreement between actual and estimated data is perfect. The positive values of RE and CE indicate the reconstruction skill in excess of climatology and negative values indicate less skill than climatology.

The calibration and verification statistics are listed in Supplementary **Table S6**, with all tests passed.
